# A prospective analysis of viral immune escape in the chronic phase of the subtype C HIV-1 infections of India

**DOI:** 10.1186/1471-2334-12-S1-P25

**Published:** 2012-05-04

**Authors:** Shilpee Sharma, Pachamuthu Balakrishnan, Suniti Solomon, Udaykumar Ranga

**Affiliations:** 1 Jawaharlal Nehru Centre for Advanced Scientific Research, HIV-AIDS Laboratory, Bengaluru, India; 2 YRG Centre for AIDS Research and Education, Chennai, India

## Background

HIV-1 is capable of evading CTL immune response through mutations in residues both within the epitopes and in sequences flanking the epitopes leading to viral diversity - a major obstacle for vaccine design. The present study aims at identifying the HLA-restricted CTL escape mutations primarily in the asymptomatic phase, and examining a correlation between such mutations and disease progression.

## Methods

In a prospective study, a cohort of select seropositive drug-naive subjects categorized into one of three clinical groups (long-term non-progressors, regular progressors and rapid progressors), based on the archived clinical records at YRG CARE, are being monitored for two years with repeated sampling at 6-month intervals. The phylogenetic analysis of the *gag* sequences was inferred using the Neighbour-Joining method in MEGA 5 to monitor viral evolution and to study CTL escape. ELISPOT will be used for mapping of the immunodominant CTL epitopes in gag with an emphasis on identifying a possible correlation between such immune responses and disease progression.

## Results

Twenty plasmid clones of *gag* have been sequenced from ten patients each at the base level. Escape mutants in several known immunodominant CTL epitopes have been identified in many of the subjects. The viral isolates phylogenetically clustered with the reference subtype C sequences. Additionally, multiple sequences from individual viral isolates clustered together indicating genetic-relatedness. Sequence analysis with REGA HIV-1 Subtyping Tool further confirmed the subtype C identity of all the viral isolates.

## Conclusion

The preliminary data are suggestive of viral escape in the chronic phase of the viral infection.

